# Summary of Prof. Yin’s CSEMV-EVCNA award lecture 2021

**DOI:** 10.20517/evcna.2022.16

**Published:** 2022-04-13

**Authors:** Ying Zhang, Hang Yin

**Affiliations:** ^1^School of Pharmaceutical Sciences, Key Laboratory of Bioorganic Phosphorous chemistry and Chemical Biology(Ministry of Education), Tsinghua University, Beijing 100084, China.; ^2^Tsinghua-Peking Center for Life Sciences, Tsinghua University, Beijing 100084, China.; ^3^Beijing Advanced Innovation Center for Structural Biology, Beijing Frontier Research Center for Biological Structure, Tsinghua University, Beijing 100084, China.

**Keywords:** Extracellular vesicles (EVs), peptide probe, detection methods

## Abstract

Extracellular vesicles (EVs) have been regarded as influential intracellular delivering parcels that possess tremendous potential because of their strict and complex secretion regulation processes. However, traditional detection methods cannot monitor the secretion of EVs due to their small particle diameters. Inspired by their peculiar diverse appearances and lipid membranes ingredients, we developed an innovative strategy to detect EVs in any kind of fluids by using rationally designed peptide probes that particularly recognize the highly curved surface of EVs. These peptide probes also serve as novel tools to selectively target cancerous cells with specific lipid compositions and distributions. With this strategy, we discovered a series of EV-secreting regulation mechanisms and identified their roles within physiological processes. Recently, we found that the transportation of oligodeoxynucleotides and cell division control protein 42 homolog from TLR9-activated macrophages to naïve cells via EVs exerts synergetic effects in the propagation of the intracellular immune response, which suggests a general mechanism for EV-mediated uptake of pathogen-associated molecular patterns.

## INTRODUCTION

Microvesicles and exosomes are two subsets of extracellular vesicles (EVs) with diameters of 30-1000 nm^[1,2]^. EVs are secreted by all living cells and then maintained in fluids or taken up by cells after being packed with certain proteins, nucleotides, metabolites, *etc.*^[3,4]^. The secretion and uptake of EVs are precisely manipulated by regulation systems under strict and highly conserved mechanisms for intercellular communication and resource exchange in normal physiology^[5,6]^. Although normal cells use EVs for intercellular communications, they also transport molecules that promote disease progression and immune system modulation, for instance, in inflammatory autoimmune diseases, cardiovascular diseases, neurodegenerative diseases, and cancer^[7,8]^. Therefore, EVs are currently considered to be promising but underexploited potential biomarkers. Large amounts of bioactive molecules such as proteins, DNA, mRNA, microRNA, lncRNA, and lipids are found in EVs, rendering them potential biomarkers for the diagnosis of carcinomas, cardiovascular disorders, autoimmune disorders, *etc.*^[7,9]^. Furthermore, understanding the functions of EVs in the physiological environment and disease progression would shed light on EVs’ mechanism as biomarkers in these diseases.

Beginning two decades ago, we focused on developing the technology for EV peptides sensing through chemical and biological tools. In conducting this work, we have sought to elucidate previously obscure regulatory mechanisms of EVs in diverse diseases. Our curvature-sensing biotechnology works by targeting protein-lipid interactions. EV-enabled biofluid diagnostics such as our curvature-sensing biotechnology are potentially applicable to tumor metastasis and other diseases. Our laboratory has also successfully developed several promising peptides and peptidomimetic agent candidates that can sense and induce membrane curvature, providing a potential measure of the concentration of exosomes in solution and blood plasma. With these tools, we have broadened the horizon for EVs’ diagnostic and treatment functions and characterized the relevant machinery that EVs use to influence the onset and progression of diseases.

### Curvature and lipid-sensing peptide probes for EV detection

It has been demonstrated that EVs exist in various kinds of body fluids, such as plasma, saliva, urine, breast milk, and cerebrospinal fluid, and that biomarkers remain stable inside the EVs because of the protection afforded by their lipid bilayers. Conventional diagnostics for cancer, such as biopsy or antinuclear antibody tests, have drawbacks, including the invasive nature of biopsies and their limitation in specificity and sensitivity. The challenges posed by conventional methods call for a breakthrough to address existing technical bottlenecks. Examination of EVs in human bodily fluid may allow for a new method of non-invasive liquid biopsy and could therefore serve as a pivotal diagnostic tool for various diseases. The development of specific, efficient, and minimally invasive probes for the detection of disease biomarkers offers a remarkable opportunity for advancing both basic, applied, and clinical research.

Nonetheless, despite the crucial role EVs could play in liquid biopsy developments, there are still problems with their efficient isolation. Current techniques for EV isolation, such as size exclusion, ultracentrifugation, immunoaffinity, or polymeric precipitation, are tedious, expensive, or have limitations for acquiring adequate EV quantities or purity. To deal with these problems, we have designed peptide probes to capture EVs by sensing their highly curved membranes and specific lipid compositions, independent of protein and oligonucleotide cargoes, thus enabling a paradigm shift from the conventional immunoaffinity approach to the universal recognition of ubiquitous phospholipids in EV membranes based on *peptide-lipid interactions*.

Diagnostic EVs are believed to be superior to some traditional methods in terms of sensitivity and speciﬁcity. More importantly, they can be collected from and detected in bodily liquids such as blood serum and urine, so their extraction is, at the most, minimally invasive^[10]^. The most recognized standard for EV isolation is ultracentrifugation, but the capital outlay cost of the instrument and the long EV isolation process (up to 12 h of continuous centrifugation) prohibit its utility in routine clinical use. New EV-based diagnostic kits have been approved by the US Food and Drug Administration for clinical use. However, there are still several limitations in exosome diagnostics. The quick, efficient, and effective isolation of EV from a bodily fluid sample is a prerequisite for their translation into clinical use^[10]^, as contaminants may affect the accuracy of the work. Newly developed techniques such as size exclusion, immunoaffinity, or polymeric precipitation are tedious, limited to specific protein recognition, or yield samples contaminated with materials that adversely affect downstream analyses. Therefore, developing more efficient and standard separation methods for clinical diagnostics is a necessity. 

Several proteins or peptides that interact with the membrane bilayer, including but not limited to Bin-Amphiphysin-Rvs domains^[11]^, Arf-GAP lipid packing sensors^[12]^, synaptotagmin I^[13]^, myristoylated alanine-rich protein kinase C substrate effector domain (MARCKS-ED)^[14,15]^, and bradykinin (BK)^[16]^, have been reported to possess lipid-binding domains. We demonstrated that the C2B loop 3 of synaptotagmin could be truncated and cyclized using solid-phase “click chemistry” to recuse its lipid and EV curvature sensing properties^[13]^. MARCKS-ED was investigated via empirical and theoretical processes to elucidate its interactions with lipid located in membrane bilayers ^[14,15]^, providing insights into the correlation of its structure and functions. We further found that the curvature sensing behavior of BK may be due to its negatively charged phosphatidylserine (PS) lipid component, which aids in its binding affinity and its lipid packing effects in smaller vesicles, and which may even allow for hydrophobic Phe interactions with the membrane bilayers. We found that the peptides’ synthetic lipid vesicle binding ability was also translated to the detection of EVs, which offers an exciting new direction in the study of cell-derived lipid vesicles that carry membrane-protected information for intercellular communications.

Previously, we showed that the monomeric and trimeric forms of BK bind on synthetic nanovesicles and EVs, with multimerization yielding an increased binding affinity of up to 7 uM^[16]^. BK (RPPGFSPFR) is a cationic peptide ligand for B_1_ and B_2_ G-protein coupled receptors. It is believed that the conformation adopted by peptide ligands such as BK is facilitated by interactions with membrane phospholipids prior to receptor binding and activation^[17,18]^. Previous studies reported that this molecule shows differential interactions with lipid vesicles^[17]^ and micelles^[19]^ and has a stronger preference for mixtures with higher anionic phospholipid composition^[17]^, which makes it an excellent candidate for capturing EVs.

The proline-induced beta-turn orients the arginine residues to a claw-like conformation that is critical for lipid recognition and attachment. Since the outer leaflets of EVs are enriched with anionic lipids as a consequence of their biogenesis^[20,21]^, it is possible that BK analogs could sense PS-enriched lipid vesicles such as EVs and work as capture and isolation agents. Moreover, the rational design of peptides can significantly alter the degree of peptide-lipid interactions. Overall, the EV-sensing peptide technique is innovative and significant because its successful implementation will offer a new approach for rapid isolation of EVs, independent of their biomolecule cargoes, and fulfill an unmet need for a simple, efficient, and high throughput EV isolation method for disease diagnosis. 

### The role of EVs in intercellular innate immune responses

Another research focus in our laboratory has been to decipher the function of EVs in response to immune stimuli. Using the above-mentioned chemical biology tools and others to detect and track EVs, we recently deciphered the mechanism of innate immunity and pro-inflammatory signaling. Sensing pathogen-associated molecular patterns and danger-associated molecular patterns is a vital step in innate immune responses. Because intracellular innate immunity has already attracted attention, we chose to investigate the role of EVs in intercellular communication and the activation of innate immunity.

**Figure 1:**
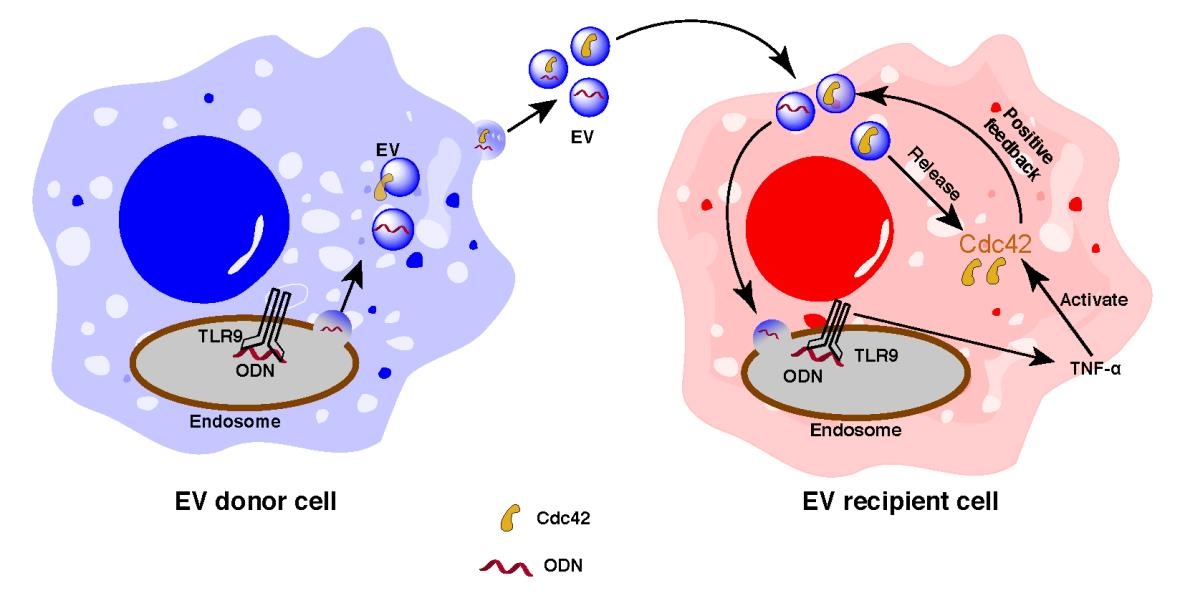
Roles of extracellular vesicles (EVs) in the communication between macrophages and propagation of the intracellular immune response (this figure is reprinted from *Science Advances* journal)^[22]^.

Endosomal Toll-like receptors (TLR) are an important family of proteins in innate immune responses capable of recognizing foreign nucleic acid sequences and then mediating innate immune responses. In our study, aided by real-time tracking of EVs, we showed that EVs transported a DNA ligand, oligodeoxynucleotides (ODN), for TLR9 activation, revealing the role of EVs in innate immune surveillance^[22]^. In addition, we found that cellular uptake of EVs is increased upon TLR9 activation, which was demonstrated by a biological tool showing real-time EV transfer between cells called the Cre-LoxP reporter system^[22]^. Further, we found that cell division control protein 42 homolog (Cdc42) is responsible for the increased cellular uptake of ODN-induced EVs from macrophages. In particular, we found that EV-carried ODN induced the release of tumor necrosis factor-α (TNF-α) from macrophages, transforming Cdc42 into its activated form, which is bound to guanosine triphosphate inside cells and accounts for the further uptake of EVs. In this study, we elucidate a new mechanism that facilitates the understanding of the positive feedback loop influenced by the uptake of EV protein cargo^[22]^. Our findings not only shed light on a new mechanism via which EVs may shuttle protein to increase their cellular uptake but also point out that Cdc42 could be a novel target for developing more efficient therapeutic approaches to regulate EV uptake [Figure 1].

### Discussion and perspectives

Here, we briefly introduce our previous work using chemical and biological tools to enable EV sensing and facilitate the understanding of the functions and mechanisms in EVs’ role in physiological or pathological conditions. Moreover, EVs have been characterized and investigated in various diseases including cancer, and this work was summarized and discussed in our recent publication^[23]^. In addition, EVs show great potential in drug delivery by acting as a Trojan horse, even penetrating biological barriers such as the blood-brain barrier. Notably, in the current pandemic situation, EVs have been approved in clinical trials as therapeutic entities and are demonstrating great potential in disease treatment, in addition to serving as biomarkers. Very soon, EVs may lead to a new era in both the pharmaceutical and biomedical fields, affecting mechanisms for the surveillance of disease, drug delivery systems, or even drug development and treatment for a variety of diseases.

## DECLARATIONS

### Copyright

© The Author(s) 2022.
